# Comparison of Mechanical Resistance to Maximal Torsion Stress in Original and Nonoriginal or Compatible Prosthetic Implant Screws: An In Vitro Study

**DOI:** 10.1155/2021/5133556

**Published:** 2021-12-02

**Authors:** António Sérgio Silva, José Manuel Mendes, Tiago Araújo, Carlos Aroso, Pedro Barreiros

**Affiliations:** ^1^Dental Science Department, Instituto de Investigação e Formação Avançada Em Ciências e Tecnologias da Saúde (IINFACTS), Rua Central da Gandra 1317, 4585-116 Gandra, Portugal; ^2^Department of Oral Rehabilitation, Instituto Universitário de Ciências da Saúde, Rua Central da Gandra 1317, 4585-116 Gandra, Portugal

## Abstract

Micromovements of the implant-abutment connection influence peri-implant bone preservation. The maximal torque after a cycle of implant prosthetic screw tightening using original components of different manufacturers and replicas produced by other companies is evaluated and quantified in this study. A total of 30 Mis Seven® standard platform implants and 30 interfaces were used, and 30 standard platform screws were tested, 10 Mis®, 10 Iconekt®, and 10 Exaktus®. The screws were tightened with an MIS® torquemeter until their respective fracture, and the fracture point was measured through the equipment's load cell, CS-Dental Testing Machine®. The screws were analyzed under an Olympus® SZ61 microscope. The fracture points were recorded and compared among all samples. To compare the mean values of the fracture torques, t-tests were performed using the reference values associated with each brand and the sample results. The variable “Place of Fracture” between the original Mis® brand and the Exaktus® replica compared to the Iconekt® replica presented a statistically significant difference (*p* < 0.001). When analyzing the variable “Fracture Torque,” although it was verified that the replica screws (Iconekt® and Exaktus®) had a lower maximum torque, 65.11 Nm and 62.89 Nm, respectively, compared to the original Mis® brand (70 Nm and 69 Nm), there were no statistically significant differences *p* > 0.05. Nonoriginal screws did not present different fracture resistances compared to the original Mis® brand screws. The fracture site of Iconekt® screws showed a different pattern compared to the other brands.

## 1. Introduction

The use of dental implants dates back approximately 1300 years to the Mayan civilization, when people replaced missing teeth with tooth-shaped shells [1].

Nowadays, with the phenomenon of osseointegration well implemented in implantology, the use of implants has been increasing, and it is possible to rehabilitate almost all types of dental absences [2, 3].

Several problems have arisen with the use of dental implants, with screw losses proving very common complications, particularly in the first designs of components for dental implants. Thus, other prosthetic systems, special screws, and torque wrenches have been investigated and developed, subsequently reducing the rates of such failures [4–6].

Regardless of the tolerance between the interface/implant parts, the screw and the level of torque used play an important role in the stability of the union when subjected to cyclical loads. The greater the torque applied to the retaining screw and prosthetic interface, the smaller the micromovement between the parts [7, 8].

The bonding force starts when the bolt is tightened using a tool. The screw head contacts the inside of the prosthetic interface as well as the screw threads with the internal threads of the implant. In the tightening process, the screw is subjected to tension and stretches, pressing the two parts against each other (prosthetic interface and implant), thus establishing what is known as the bonding force [9–11].

Loosening or fractures of the prosthetic interface screw can present a severe complication, as it is necessary to remove the restoration to gain access to the screw. As a result, cemented restorations can be damaged or destroyed in this process [12].

There is no consensus in the literature on screw loosening, with reports of loosening rates ranging from 2% to 40%. The most likely cause of most screw loosening is improper screw tightening [13, 14].

Torque control is the primary method used in dentistry for tightening prosthetic interface screws, and manufacturers specify the torque at which screws need to be tightened to achieve an optimal preload [15, 16].

The longevity and success of implant rehabilitations are related not only to the stability of the implant but also to a correct and efficient union between the prosthetic interface and the implant, with the prosthetic screw being a crucial element in maintaining this union [17, 18].

The aim of this study is to evaluate the fracture resistance of original and nonoriginal screws, as well as to verify their fracture pattern, clarifying if the use of nonoriginal brands has the same clinical performance when compared with original brands, in respect to resistance to maximum torsion.

## 2. Materials and Methods

### 2.1. Sample Characteristics

A total of 30 Mis Seven® standard platform implants and 30 interfaces were used, and 30 standard platform screws were tested, 10 Mis®, 10 Iconekt®, and 10 Exaktus®.

**Figure 1:**
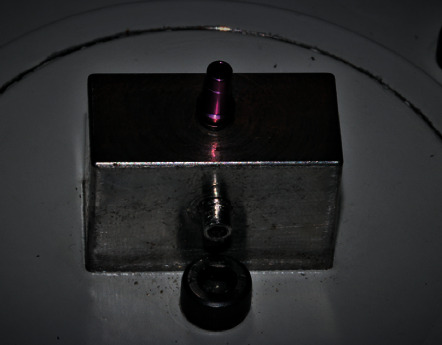
Prosthetic interface fixed in the test base.

The implants with the prosthetic interface fixed with the screw to be tested were mounted on a base and correctly stabilized (Figure 1).

**Figure 2:**
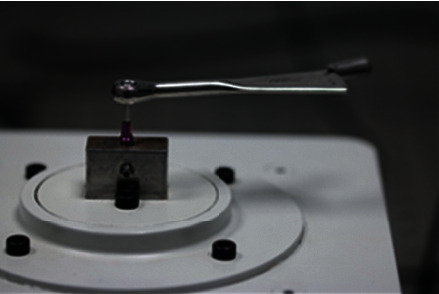
Representation of the torquemeter assembly.

**Figure 3:**
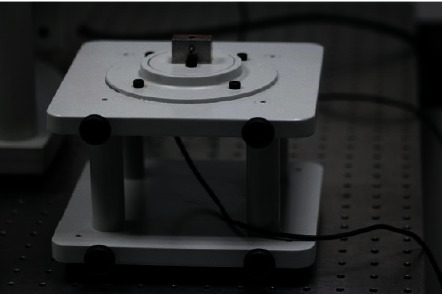
CS-Dental Testing Machine® load cell.

The screws to be tested were subjected to a torsional force and manual tightening with an MIS torquemeter (Figure 2) until their respective fracture, with the fracture point measured using the load cell (Figure 3).

**Figure 4:**
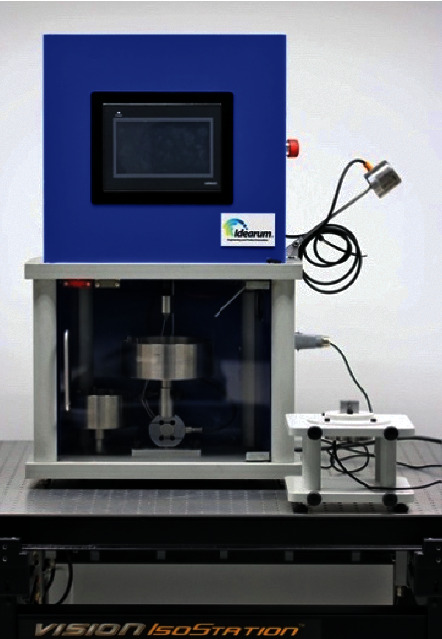
CS-Dental Testing Machine® (Barcelona, Spain).

The equipment used for the study was a CS-Dental Testing Machine® (Barcelona, Spain), model ID1-BAD (Figure 4). The CS-Dental Testing Machine® (Barcelona, Spain) is a fatigue test device built in compliance with the 2006/42/CE safety of machines and the norms EN 12100–1/2, EN 954–1, EN 1037, EN 61310–1/2, EN 60204–1, EN ISSO 14121–1, and EN ISSO 13850.

The screws to be tested were subjected to a torsional force with a torque wrench until fracture occurred. A brand-new torque wrench was also used every time a prosthetic screw was tested. The fracture points were automatically recorded in the machine and compared.

The screws were analyzed under an Olympus® SZ61 (Tokyo, Japan) microscope to view the fracture pattern at the macroscopic level.

### 2.2. Data Collection

A standard laboratory protocol was established and applied at the Institute for Research and Advanced Training in Health Sciences and Technologies (IINFACTS-CESPU) to test all the selected samples.

All prosthetic screws from the manufacturers were labeled with a serial number and control date: 10 Mis® prosthetic screws, 10 Iconekt® prosthetic screws, and 10 Exaktus® prosthetic screws.

The presence of anomalies and defects was assessed with a stereoscope (Olympus® SZ61-Tokyo, Japan), and a 90× magnifier was used to evaluate any changes in the surfaces.

The prosthetic abutment was coupled to the implant analog (of the corresponding brands) with a prosthetic screw with a respective manual key. The prosthetic screw was tightened by hand until there were no gaps between the two parts. A brand-new manual screwdriver was used every time a prosthetic screw was tested.

The two parts were placed in a load cell and connected to the CS-Dental Testing Machine®.

The screws to be tested were subjected to a torsional force with a torque wrench until fracture occurred. A brand-new torque wrench was also used every time a prosthetic screw was tested.

The fracture points were automatically recorded in the machine and compared between all samples.

The fractured parts were analyzed under an optical microscope to observe microscopic fracture characteristics.

### 2.3. Data Analysis

For the organization and description of the data used, descriptive statistics was used. Absolute and relative frequencies were used for the analysis of variables.

The Kolmogorov–Smirnov test was used to verify the existence of normal distribution of continuous variables. For continuous variables without normal distribution destruction, the Kruskal–Wallis test was used to verify statistical differences. In the case of categorical variables, the chi-square test was used.

Statistical analysis was performed using IBM SPSS® (Chicago, IL, USA) version 22.0 software.

## 3. Results

**Figure 5:**
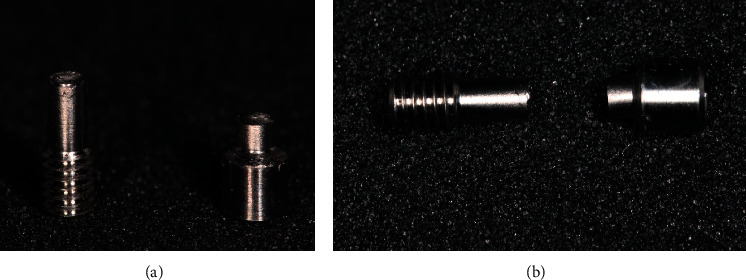
(a), (b) Fracture pattern of MIS® screws.

After performing the mechanical stress tests on the screws, it was found that, in the MIS® brand, 9 of the 10 screws tested fractured, and all fractures occurred in the arm area (Figures 5(a) and 5(b)). The average fracture torque was 70.69 Ncm with a standard deviation of 6.6 Ncm.

**Figure 6:**
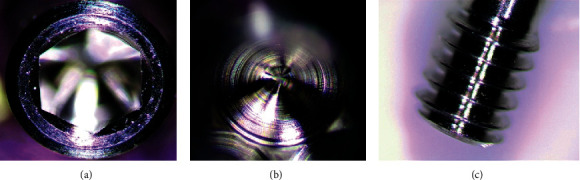
MIS® screw magnification analysis: (a) screw head, (b) inside screw connection, and (c) screw thread region.

In the macroscopic analysis using a 90× magnification, it was found that the screw had a clean finish (Figure 6(a)) with no chips inside the screw head (Figure 6(b)) or in the thread region (Figure 6(c)).

**Figure 7:**
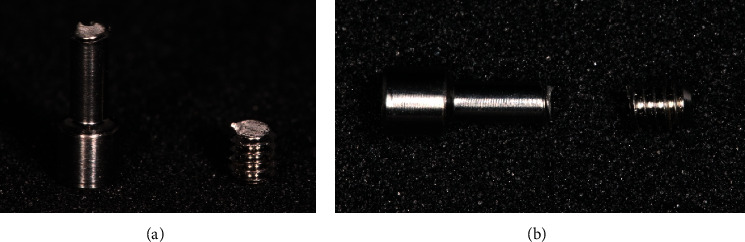
(a), (b) Fracture pattern of Iconekt® screws.

In the Iconekt® brand, 8 of the 10 screws tested fractured, and all the fractures occurred in the first turn of the thread (Figures 7(a) and 7(b)).

The mean fracture torque was 65.11 Ncm with a standard deviation 11.02 Ncm.

**Figure 8:**
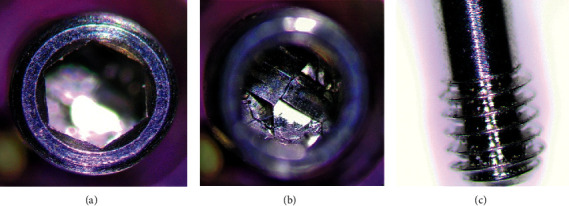
Iconekt® screw magnification analysis: (a) screw head, (b) inside screw connection, and (c) screw thread region.

Upon observation with the microscope, there was a clean finish on the outside of the screw head (Figure 8(a)); swarf can be observed inside the screw head (Figure 8(b)) and also in the thread area (Figure 8(c)).

**Figure 9:**
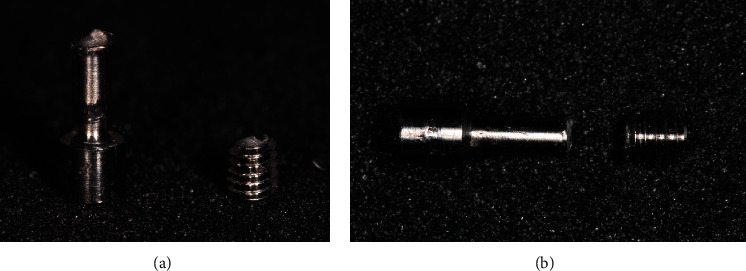
(a), (b) Fracture pattern of Exaktus® screws.

In the Exaktus® brand, all the 10 screws tested fractured: nine in the thread area of the first turn and one in the arm (Figures 9(a) and 9(b)).

The mean fracture torque was 62.89 Ncm with a standard deviation of 5.78 Ncm.

**Figure 10:**
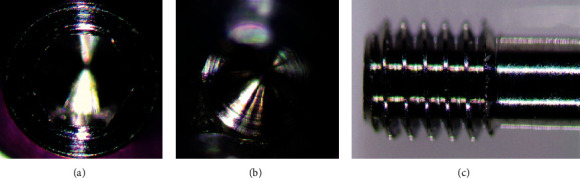
Exaktus® screw magnification analysis: (a) screw head, (b) inside screw connection, and (c) screw thread region.

Upon observation with the microscope, it was verified that the screw had a clean finish (Figure 10(a)) with no chips inside the screw head (Figure 10(b)); in the thread region, there were small traces of chips (Figure 10(c)).

In the statistical analysis, the variables “fracture torque” and “fracture site” were studied. The variable “fracture torque” in the various brands did not have a normal distribution, so a non-parametric Kruskal–Wallis test was used, and there were no statistically significant differences in the means of fracture torque between the various brands.

In the analysis by fracture site, the chi-square test was used, and there were statistically significant differences (< 0.001) between the MIS® and Iconekt® brands.

## 4. Discussion

With the use of dental implants with screw-retained prosthetic rehabilitations, a correct torque on the screw is essential so that an ideal preload occurs in the implant union-prosthetic interface.

Loosening or fracture of the prosthetic screw is related to an implant-prosthetic interface mismatch, and the presence of a gap between the implant and the prosthetic interface can cause unfavorable stress distributions within the connection components, the implant, and the bone. Screw loosening or fractures are common complications in implant restorations. It is speculated that the gap between the implant and the prosthetic interface has a significant influence on these findings [14]. Many authors report that screw loosening is one of the most common prosthetic complications in rehabilitation with implants, and that it may be related to the tightening technique or insufficient torque when tightening. Some authors have reported that the higher the torque and the higher the preload, the lower the probability of screw loosening and consequent detachment of the prosthetic interface [8].

According to the work of Piermatti et al. [15], the strength of the screw material has a significant influence on the preload. Typically, bolted connection manufacturers recommend tightening to 75% to 80% of the material's yield strength to avoid permanent deformation. However, the stronger the bolt, the greater the preload that can be achieved. This is only partially true, as after the screw is tightened beyond a certain point, the friction between the implant and screw threads becomes so great that the screw head hexagon dusts or the installation wrench fractures. In our study, there was no fracture of the installation wrench, but three screw heads became worn upon tightening (1 from MIS®, 2 from Iconekt®); in the Exaktus® brand, there was no dusting. Additionally, according to these authors, an aspect that should be considered for reducing screw loosening is the application of higher torque values. In current bolt designs, torque values of 40 or even 50 Ncm can be applied without incurring plastic deformation. In our study, it was found that a screw fractured below the values described by these authors (41.58 Ncm, Iconekt®). The use of higher torque values could increase the preload and would provide increased connection separation strength and greater bolt stability.

The aim of this study was to test the same screws, but from different manufacturers, with the originals and replicas made by other companies. The material used in the manufacture of the screws was the same in all brands studied: titanium 6Al4 V.

There are no previous studies comparing original and replica screws in the field of dentistry. The results obtained show statistically significant differences when analyzing the variable “Place of Fracture” between the original Mis® brand and the Exaktus® replica compared to the Iconekt® replica.

In the 10 Mis® screws and in 9 of the 10 Exaktus® screws tested, the fracture site was in the arm, whereas for Iconekt®, all 10 screws fractured in the thread area first. These results are surprising since the material they are made of and the morphologies are similar between the brands. Further studies are needed to determine if there is any property in the alloy or in the morphology of the screws that could give rise to this outcome.

When analyzing the variable “fracture torque,” although it was verified that the replica screws (Iconekt® and Exaktus®) had a lower maximum torque, 65.11 Ncm and 62.89 Ncm, respectively, compared to the original Mis® brand, 70 Ncm and 69 Ncm, there were no statistically significant differences.

## 5. Conclusions

In the present study, we conclude that since no statistically significant differences were found, Iconekt® and Exaktus® replica screws do not present different fracture resistance when compared to MIS® brand screws.

Although not the central point of the study but a pertinent analysis nonetheless, it was found that the fracture site of Iconekt® brand screws presented a different pattern from the other brands, with statistically significant differences. The MIS® screws fractured in the screw arm region, the Exaktus® screws also fractured in the screw arm region, and the Iconekt® screws fractured in the thread region.

This finding merits further study, either with a possible evaluation of the chemical composition of the materials of the screws or a more detailed analysis of the design of the screws and their comparison; a larger sample could permit identification of a statistically expressed difference regarding the fracture torque.

## Data Availability

The data that support the findings of this study are available from the corresponding author upon reasonable request.
